# Enhanced Sampling for Efficient Learning of Coarse-Grained Machine Learning Potentials

**DOI:** 10.1021/acs.jctc.5c01712

**Published:** 2025-12-24

**Authors:** Weilong Chen, Franz Görlich, Paul Fuchs, Julija Zavadlav

**Affiliations:** † Professorship of Multiscale Modeling of Fluid Materials, Department of Engineering Physics and Computation, TUM School of Engineering and Design, 9184 Technical University of Munich, Munich 80333, Germany; ‡ Atomistic Modeling Center (AMC), Munich Data Science Institute (MDSI), Technical University of Munich, Garching 85748, Germany

## Abstract

Coarse-graining (CG) enables molecular dynamics (MD) simulations of larger systems and longer time scales that are otherwise infeasible with atomistic models. Machine learning potentials (MLPs), with their capacity to capture many-body interactions, can provide accurate approximations of the potential of mean force (PMF) in CG models. Current CG MLPs are typically trained in a bottom-up manner via force matching, which in practice relies on configurations sampled from the unbiased equilibrium Boltzmann distribution to ensure thermodynamic consistency. This convention poses two key limitations: first, sufficiently long atomistic trajectories are needed to reach convergence; and second, even once equilibrated, transition regions remain poorly sampled. To address these issues, we employ enhanced sampling to bias along CG degrees of freedom for data generation and then recompute the forces with respect to the unbiased potential. This strategy simultaneously shortens the simulation time required to produce equilibrated data and enriches sampling in transition regions, while preserving the correct PMF. We demonstrate its effectiveness on the Müller-Brown potential and capped alanine, achieving notable improvements. Our findings support the use of enhanced sampling for force matching as a promising direction to improve the accuracy and reliability of CG MLPs.

## Graphical abstract

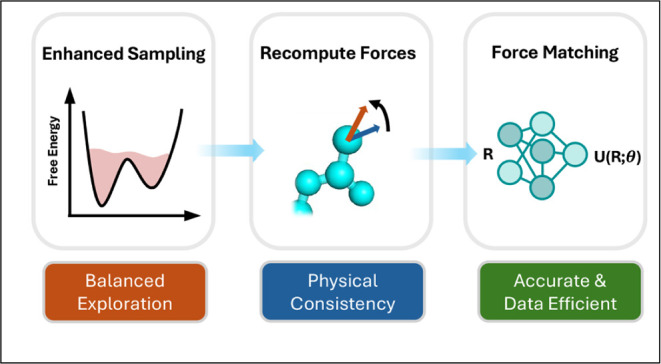

## Introduction

Molecular Dynamics (MD) simulations play an important role in science and engineering, providing access to a wide range of structural, dynamical, and thermodynamic properties of molecular systems.^1,2^ In statistical mechanics, such observables can be expressed in terms of expectation values with respect to a statistical distribution (*ensemble*) over microscopic states, defined by macroscopic control parameters such as temperature, volume, pressure, and chemical potential.^3^ For example, the canonical Boltzmann distribution, $p\left(r\right)=Z^{-1}\exp\left(-u\left(r\right)/k_{B}T\right)$ describes the *NVT* ensemble, in which temperature (*T*), volume (*V*), and particle number (*N*) remain constant. For molecular systems, the high dimensionality of configuration space makes direct evaluation of the partition function Z intractable. In practice, sampling based methods such as Markov Chain Monte Carlo (MCMC) or MD^4^ are used to generate configurations from the target distribution. However, the rugged free energy landscapes characteristic of many molecular systems lead to slow decorrelation between samples, making it necessary to run prohibitively long simulations to obtain independent statistics. As a result, extensive sampling of large macromolecular complexes on relevant time scales remains beyond the reach of atomistic resolution.

To address this challenge, a variety of enhanced sampling methods have been developed.^5^ These approaches accelerate the exploration of the configuration space either by modifying the statistical ensemble to promote rapid transitions between free energy basins or by coupling simulations across multiple thermodynamic ensembles (*replicas*).^6,7^ A notable family of approaches are biasing methods, which perform importance sampling by applying a bias potential that can be reweighted to recover unbiased ensemble statistics.^8,9^ The bias potential can be static, as in umbrella sampling,^10^ or updated dynamically, as in metadynamics,^11^ and is typically defined in terms of a small set of collective variables that capture the slow degrees of freedom of the system.

Coarse-graining (CG) offers a complementary approach by simplifying atomistic models into reduced representations that capture essential interactions.^12–14^ This reduction in dimensionality smooths the free energy surface and decreases computational cost, thus extending the time and length scales accessible to simulation^15^ while also improving the statistical efficiency of the reweighting procedures. In many applications,^16,17^ enhanced sampling and coarse-graining can be combined, allowing researchers to combine the benefits of both methods to efficiently explore complex molecular systems.^18^

Traditional CG models have historically been parametrized using either ″top-down″ or ″bottom-up″ approaches. In top-down approaches, the model parameters are adjusted to reproduce macroscopic observables, such as experimental measurements. A well-known example is the MARTINI model, which was designed to reproduce experimental partition coefficients.^15^ In bottom-up approaches,^19^ the primary goal is to construct a CG model that reproduces the equilibrium configurational distribution (or free energy landscape) of the fine-grained system.^12^ While this ensures the model captures correct static correlations, it does not guarantee the accurate reproduction of dynamical properties.^12,19^ By construction, the exact CG potential is the many-body potential of mean force (PMF). However, traditional attempts to approximate the PMF using functional forms or large basis sets similar to classical all-atom potentials have generally proven limited, as added complexity rarely guarantees improved accuracy or transferability.^12^

Deep learning has opened new avenues for equilibrium sampling of CG systems.^20^ An important direction is the development of CG machine learning potentials (MLPs),^21,22^ which aim to learn the CG PMF,^23,24^ analogous to the potential energy function in atomistic systems. These models are typically trained using bottom-up approaches such as variational force matching^25,26^ (FM) or relative entropy minimization.^27^ In FM, the model is trained to minimize the mean squared error between the predicted CG forces and the atomistic forces projected onto the CG space. However, MLPs often depend on prior potentials to ensure reliable predictions outside the training domain, and the corresponding free energy surface is sensitive to errors in transition regions. Relative entropy minimization provides an alternative but is computationally more expensive due to the requirement of repeated simulations during training.^28,29^ Recent work has also focused on improving the accuracy and transferability of CG MLPs.^30–37^

Another active line of research explores deep generative models for CG systems.^38–44^ Boltzmann Emulators,^45–47^ for example, act as surrogate models by learning a biased distribution that enables one-shot sampling. The connection between generative models and molecular dynamics has led to new sampling approaches. For instance, Flow-Matching^48^ improves data efficiency by training a normalizing flow to approximate the target distribution and then derives forces from the generated samples to train a CG MLP. This shares the goal of relative entropy minimization in reproducing the target distribution, but circumvents the need for iterative CG simulations. Diffusion models provide another approach by directly estimating forces via the score function to enable CG MD simulations.^49–53^ Despite these advances, generative CG models face limitations: the lack of an explicit energy function prevents unbiased reweighting, scaling to larger systems remains difficult,^54–59^ and training generally requires unbiased CG MD data. Energy-based models offer an alternative,^60^ since they do not rely on training samples, but typically require a reliable energy predictor,^61–65^ which in practice depends on the availability of existing CG MLPs.^29^

In this work, we revisit a central limitation of variational force matching for coarse-graining: the mean force can only be approximated statistically through microscopic forces with large fluctuation.^19^ In practice, FM relies on long unbiased trajectories, which are computationally demanding and yield samples concentrated around metastable states, with insufficient coverage of transition regions.^12,14,23–25^ Consequently, even highly flexible CG potentials trained using FM may perform poorly outside the stable basins and struggle to capture the correct relative probabilities of metastable states.^21,29^ To overcome these challenges, we introduce enhanced sampling methods for efficient data generation. We show that applying a bias along coarse-grained coordinates and recomputing forces with respect to the unbiased atomistic potential leaves the conditional mean force unchanged. This permits training directly on biased trajectories (without reweighting), substantially accelerating convergence while also improving coverage of transition states. We demonstrate the effectiveness of this approach on the Müller-Brown potential and capped alanine solvated in explicit water. Taken together, our results establish enhanced sampling as a powerful and general framework for constructing accurate and data-efficient CG MLPs, offering fundamental improvements over existing methods.

## Theory and Methods

The coarse-grained modeling begins with the definition of a mapping from the atomistic (AT) description to a reduced set of CG variables. Denote the AT coordinates as $r\in R^{3n}$ and the CG coordinates as $R=\xi \left(r\right)\in R^{3N}$ , with *N* < *n*. The mapping ξ groups atoms into beads, reducing dimensionality while providing a basis for constructing effective interactions that reproduce microscopic behavior. In this work, we focus on equilibrium thermodynamics in the canonical (*NVT*) ensemble and assume a linear and orthogonal mapping. Extensions to nonlinear mappings, nonequilibrium systems, and kinetic modeling have also been studied.^66–68^

To preserve the equilibrium distribution, the central requirement for a CG model is thermodynamic consistency: the equilibrium distribution of the CG system must reproduce the equilibrium distribution of the underlying AT system projected onto the CG variables. For canonical ensemble, the AT equilibrium distribution is given by

1 $$p_{\mathrm{AT}}\left(r\right)=Z^{-1}\exp\left(-\frac{u\left(r\right)}{k_{B}T}\right)$$

where *u*(**r**) is the AT potential, *k*_*B*_ is the Boltzmann constant, *T* is the temperature, and $Z=\int \exp\left(-u\left(r\right)/k_{B}T\right)dr$ is the partition function. The CG equilibrium distribution is obtained by marginalizing over the atomistic degrees of freedom,

2 $$p_{\mathrm{CG}}\left(R\right)=\int \delta \left(R-\xi \left(r\right)\right)p_{\mathrm{AT}}\left(r\right)dr$$

The exact many-body potential of mean force (PMF) U*­(**R**) is defined by the relation:

3 $$U^{*}\left(R\right)=-k_{B}T\ln p_{\mathrm{CG}}\left(R\right)+C$$

where *C* is an arbitrary additive constant. Since exact marginalization is generally intractable for complex systems, a parametric model *U*(**R**; θ) is typically learned to approximate the exact PMF. The parameters θ are determined by minimizing a variational objective, such as force matching or relative entropy minimization, such that *U*(**R**; θ) ≈ U*­(**R**).

### Force Matching

Variational force matching, also known as multiscale coarse-graining,^27^ is a commonly used approach to learn the CG MLP *U*(**R**; θ). The central idea is that the CG forces predicted by the model should match the instantaneous atomistic forces projected onto the CG coordinates, ξ­(**f**(**r**)). The FM loss is defined as the mean squared error between the projected AT forces and the predicted CG forces:

4 $$\chi^{2}\left(\theta \right)=\langle ∥\xi \left(f\left(r\right)\right)+\nabla U\left(\xi \left(r\right);\theta \right){∥}^{2}\rangle_{r}$$

where the average is taken over the equilibrium AT distribution.

The FM loss can be further decomposed into two terms,^23,24,27^

5 $$\chi^{2}\left(\theta \right)=\underset{\mathrm{PMF error}}{\underset{︸}{\langle ∥F\left(R\right)+\nabla U\left(R;\theta \right){∥}^{2}\rangle_{R}}}+\underset{\mathrm{irreducible}}{\underset{︸}{\mathrm{Noise}\left(\xi \right)}}$$

where **F**(**R**) = ⟨ξ­(**f**(**r**))⟩_**r**|**R**_ is the *mean force* conditioned on the CG state. The first term measures the deviation between the mean force **F**(**R**) and the CG forces predicted by the CG potential. The second term, Noise­(ξ), represents the irreducible variance of the projected atomistic forces arising from the many-to-one nature of the mapping ξ. This noise term depends only on the choice of mapping and cannot be reduced by optimizing the CG model. Hence, the machine learning task in FM is to find a potential *U*(**R**; θ) that best approximates the mean force **F**(**R**). For this reason, *U* is often referred to as the *potential of mean force* (PMF). Minimizing χ^2^(θ) ensures that the learned potential approximates the PMF as closely as possible given the chosen CG mapping and available data. In practice, given a finite data set of *M* atomistic configurations $D=\left\{r_{1},...,r_{M}\right\}$ , the empirical FM loss can be estimated as

6 $${\mathrm{\chi ̂}}^{2}\left(\theta \right)=\frac{1}{3M}\overset{M}{\underset{i=1}{\sum }}∥\xi \left(f\left(r_{i}\right)\right)+\nabla U\left(\xi \left(r_{i}\right);\theta \right){∥}^{2}$$

where $\xi \left(D\right)={[\xi (r_{1}),...,\xi (r_{M})]}^{T}\in R^{M\times 3N}$ and $\xi \left(f\left(D\right)\right)={[\xi (f(r_{1})),...,\xi (f(r_{M}))]}^{T}\in R^{M\times 3N}$ .

### Finite Data Size Effects

Learning CG MLPs under the force matching (FM) framework is fundamentally limited by *finite data size effects*. Two main factors contribute to this challenge.

The first arises from the nature of CG force matching. Unlike atomistic MLPs, where potential energy labels are directly available, the CG PMF *U*(**R**) must be inferred indirectly from instantaneous forces by minimizing the variational bound in eq 4. The true mean force **F**(**R**) is defined as the average of all atomistic configurations corresponding to the same coarse-grained state **R**, while a single projected force represents only one noisy sample of this average. Accurate approximation of this mean requires dense sampling in the neighborhood of each **R**, so that statistical noise does not dominate the learning signal. As a result, FM-trained CG models generally require much larger data sets than atomistic models trained on explicit energy surfaces. Although conditional averages could, in principle, be obtained using constrained MD or Blue Moon sampling,^69,70^ performing such targeted sampling for a sufficiently dense set of coarse-grained configurations is computationally prohibitive.

The second factor stems from how CG FM data sets are generated in practice. To ensure thermodynamic consistency, configurations are typically sampled from the unbiased equilibrium Boltzmann distribution of the atomistic system. Producing sufficiently long atomistic trajectories is necessary to achieve convergence of the mean forces, which can be particularly challenging for complex biomolecular systems due to rare events and slow transitions.^1^ Even once equilibrium is reached, the samples are unevenly distributed: Configurations are concentrated near metastable states, whereas transition regions remain poorly represented. This uneven sampling reduces the accuracy of the mean-force approximation in less-populated regions of configuration space.

### Unbiased Mean Forces from Biased Sampling

As we describe, a major limitation of CG force matching is the practical difficulty of generating sufficiently representative equilibrium data. Transition regions, rarely visited in standard MD, are particularly underrepresented, resulting in noisy mean force estimates and less reliable CG MLPs. Enhanced sampling methods, such as umbrella sampling, metadynamics, or other biasing strategies, are natural choices to improve coverage of these regions. A natural question arises: does training on biased data distort the CG mean force and thereby compromise thermodynamic consistency?

We formalize this question as follows. Let *W*(**R**) denote a bias potential applied along the CG coordinates. The biased AT distribution is given by

7 $$p_{W}\left(r\right)=Z_{W}^{-1}\exp\left(-\beta \left(u\left(r\right)+W\left(\xi \left(r\right)\right)\right)\right)$$

The key observation is that although the bias changes the marginal distribution of the CG coordinates, it does not alter the conditional distribution of atomistic configurations at fixed **R**. The conditional distribution under the bias is

8 $$p_{W}\left(r|R\right)=\frac{\delta \left(\xi \left(r\right)-R\right)e^{-\beta (u(r)+W(R))}}{\int \delta \left(\xi \left(r\right)-R\right)e^{-\beta (u(r)+W(R))}dr}$$

Since *W*(**R**) is constant when conditioning on **R**, it cancels between numerator and denominator:

9 $$p_{W}\left(r|R\right)=\frac{\delta \left(\xi \left(r\right)-R\right)e^{-\beta u(r)}}{\int \delta \left(\xi \left(r\right)-R\right)e^{-\beta u(r)}dr}=p\left(r|R\right)$$

Thus, any bias that depends exclusively on the CG variables leaves the atomistic conditional ensemble at fixed **R** unchanged. Consequently, the coarse-grained mean force, defined as the conditional expectation of the projected forces, remains invariant:

10 $$F\left(R\right)=\langle \xi \left(f\left(r\right)\right)\rangle_{r|R}=\langle \xi \left(f\left(r\right)\right)\rangle_{r_{W}|R}$$

Intuitively, the bias alters how frequently a given CG configuration **R** is visited, but once **R** is fixed, the conditional distribution of atomistic microstates consistent with **R** is unaffected by *W*. In practice, however, the invariance derived in eq 10 relies on several underlying assumptions, outlined below:

1 **Functional Dependence of the Bias:** The bias potential must depend exclusively on the CG variables, i.e., *W* = *W*(ξ­(**r**)). Dependence on orthogonal degrees of freedom would perturb the conditional ensemble *p*(**r**|**R**), rendering the sampled mean forces thermodynamically inconsistent. In such cases, explicit reweighting is required to recover unbiased mean forces.
2 **Conditional Ergodicity:** The system needs to remain ergodic in the orthogonal degrees of freedom. While the bias accelerates the exploration of **R**, the fast degrees of freedom (e.g., vibrations, solvent) are assumed to equilibrate rapidly to the conditional Boltzmann distribution. Hysteresis or trapping in these hidden variables would yield nonequilibrium force estimates.
3 **Recovery of Unbiased Forces:** The training targets must be the forces derived from the unbiased potential *u*(**r**). The biasing force contribution, -∇_**r**_ *W*(ξ­(**r**)), must be subtracted from biased forces.

Subject to the assumptions above, the optimization over the biased distribution targets the identical mean force as the unbiased case. The biased objective function is defined as

11 $$\chi_{W}^{2}\left(\theta \right)=\langle ∥\xi \left(f\left(r\right)\right)+\nabla U\left(\xi \left(r\right);\theta \right){∥}^{2}\rangle_{r_{W}}$$

While the scalar value of the loss $\chi_{W}^{2}$ differs from the unbiased objective χ^2^ due to the modified sampling density, both functionals share the same global minimum with respect to θ (see Supporting Information for a detailed proof). This invariance enables the training of CG potentials on data sets generated with biased sampling, without requiring reweighting of the loss function. The practical advantages are 2-fold: (i) biased simulations accelerate exploration of rarely visited states, reducing the total simulation time needed for data generation, and (ii) the resulting data sets provide more uniform coverage of both energy basins and transition regions, leading to more accurate and robust CG MLPs. An overview of the enhanced sampling force matching workflow is shown in Figure 1.

**Figure 1:**
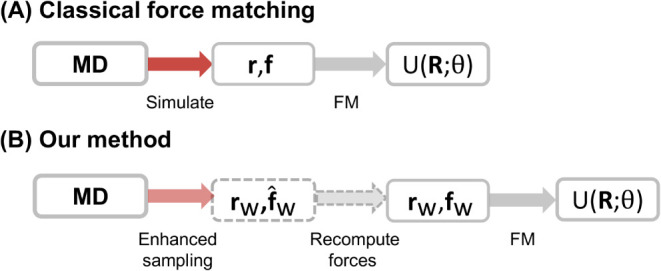
Overview of the enhanced sampling force matching method. (A) Classical force matching: positions **r** and forces **f** from an unbiased atomistic MD simulation are used to learn the potential of mean force (PMF) *U*. (B) Enhanced sampling force matching (this work): Configurations are obtained via enhanced sampling, reducing the required simulation time (light red region). The forces acting during the biased simulation are **f̂**_*W*_(**r**_*W*_) = -∇_**r**_(*u*(**r**_*W*_) + *W*(ξ­(**r**_*W*_))), while the unbiased forces used for training are recomputed using the unbiased potential, **f**_*W*_(**r**_*W*_) = -∇_**r**_ *u*(**r**_*W*_), which incurs minimal additional computational cost. The PMF is learned from the biased configurations **r**_*W*_ and their corresponding recomputed forces **f**_*W*_.

### Enhanced Sampling Methods

The invariance of mean forces under CG coordinate-dependent biasing (eq 10) shows that biased simulations can be directly used for force matching, provided that forces are recomputed with respect to the unbiased atomistic potential. This observation allows us to incorporate enhanced sampling methods that accelerate exploration of rarely visited or high-barrier regions. Here, we focus on two popular choices: umbrella sampling and well-tempered metadynamics.

#### Umbrella Sampling

Umbrella sampling^10^ improves sampling efficiency by applying a static bias potential *W*(**R**) that confines the system near a chosen region of the (CG) coordinate space. In this work, we employ a single harmonic constraint centered on **R**_0_,

12 $$W\left(R\right)=\frac{1}{2}\kappa ∥R-R_{0}{∥}^{2}$$

where κ is the force constant. The biased AT distribution is

13 $$p_{W}\left(r\right)\propto \exp\left(-\beta \left(u\left(r\right)+W\left(\xi \left(r\right)\right)\right)\right)$$

This ensures better sampling of the configurations around **R**_0_, allowing a better representation of transition regions that are otherwise rarely observed in unbiased trajectories.

#### Well-Tempered Metadynamics

Metadynamics^11^ enhances sampling by progressively filling free energy basins with a history-dependent bias, thereby discouraging revisiting previously explored regions. At time intervals τ, Gaussians of width σ and initial height *h* are deposited along the chosen CG (or CV) coordinates,

14 $$W_{t}\left(R\right)=\underset{\tau <t}{\sum }h\exp\left(-\frac{∥R-R\left(\tau \right){∥}^{2}}{2\sigma^{2}}\right)$$

In plain metadynamics, the bias keeps growing indefinitely, eventually flattening the free energy surface. Well-Tempered metadynamics^71^ improves this by tempering the Gaussian heights with a bias factor γ > 1,

15 $$W_{t}\left(R\right)=\underset{\tau <t}{\sum }h\exp\left(-\frac{∥R-R\left(\tau \right){∥}^{2}}{2\sigma^{2}}\right)\exp\left(-\frac{W_{\tau }\left(R\right)}{k_{B}T\left(\gamma -1\right)}\right)$$

In the long-time limit, this yields sampling from a modified distribution,

16 $$p_{\mathrm{WT}}\left(R\right)\propto \exp\left(-\frac{\beta }{\gamma }A\left(R\right)\right)$$

where *A*(**R**) is the free energy surface of the CG (or CV) coordinates R. This corresponds to sampling at an effective temperature T* = γ*T*, since the bias factor γ = T* /*T* rescales the thermal fluctuations along **R**. Physically, this implies that the coarse-grained variables explore the landscape as if coupled to a high-temperature heat bath at T*, effectively reducing barrier heights by a factor of 1/γ. Note that the remaining atomistic degrees of freedom (e.g., solvent, fast vibrations) remain at the physical temperature *T*. This allows the system to overcome high-energy barriers associated with the collective variables without inducing the unphysical structural denaturation that would occur in a global high-temperature simulation. The term “well-tempered” reflects the fact that the bias is added more slowly over time, striking a balance between the exploration of new regions and the preservation of meaningful free energy differences.

A practical challenge in applying enhanced sampling to novel systems is selecting bias parameters without prior knowledge of the free-energy landscape. Importantly, our method prioritizes achieving sufficient exploration of the conformational space rather than the exact convergence of the bias potential required for quantitative free-energy reconstruction. This distinction makes the method more robust to suboptimal parameter choices. For novel systems, we recommend an iterative refinement strategy. Initial parameters can be selected based on standard heuristics:^71–73^ for solvated biomolecules, the bias factor is typically chosen in the range γ ∈ [5, 20] to adequately reduce expected barriers, while the Gaussian width σ is estimated from natural thermal fluctuations observed in short unbiased precursor runs (e.g., σ ≈ 1/3).^74^ Sampling quality is then assessed by monitoring the histogram of the collective variables. If unsampled regions or “gaps” persist along transition pathways, the bias strength can be increased to help bridge these regions.

### Graph Neural Network Potentials without Priors

To parametrize the CG potential *U*(**R**; θ) for molecular systems, we adopt the MACE architecture,^75^ an equivariant message-passing graph neural network originally developed for atomistic potentials. Each CG bead is represented as a node in a graph, and edges indicate neighbor pairs within a cutoff radius and carry distance/relative-vector embeddings. Equivariant message passing layers update node features while enforcing that *U*(**R**; θ) is invariant under rigid translations and rotations and that internal vector features transform equivariantly. The force on bead *i* is obtained directly from the learned potential by automatic differentiation,

17 $$F_{i}\left(R;\theta \right)=-\nabla_{R_{i}}U\left(R;\theta \right)$$

ensuring that forces are conservative by construction.

A common strategy in the literature is to augment CG MLPs with a physics-based prior, such as baseline pairwise interactions and harmonic bonded terms, so that the network only learns a corrective energy term, which is helpful for data efficiency and improves simulation stability. In contrast, here we train MACE directly on the force-matching loss (eq 4) without including any prior.^76^ This choice avoids introducing modeling bias and allows the network to learn the PMF purely from data.

## Results

We evaluate the performance of our methods by applying them to study two representative systems: a Low-dimensional Müller-Brown Potential and MD simulation data of capped alanine in water. More detailed information on both systems can be found in the Supporting Information. For both systems, we apply enhanced sampling by introducing suitable bias potentials to promote exploration of rarely visited states. The biased forces are recomputed at each sampled configuration with respect to the unbiased potential and serve as training data for the CG MLPs. We evaluate the methods in terms of data efficiency and model accuracy. Specific hyperparameter choices for all our experiments can be found in the Supporting Information.

### Low-Dimensional Müller–Brown Potential

We consider the two-dimensional Müller-Brown (MB) potential, a canonical test system for transition path sampling,^77^ which features a global minimum and two local minima separated by saddle points (Figure 2A).

**Figure 2:**
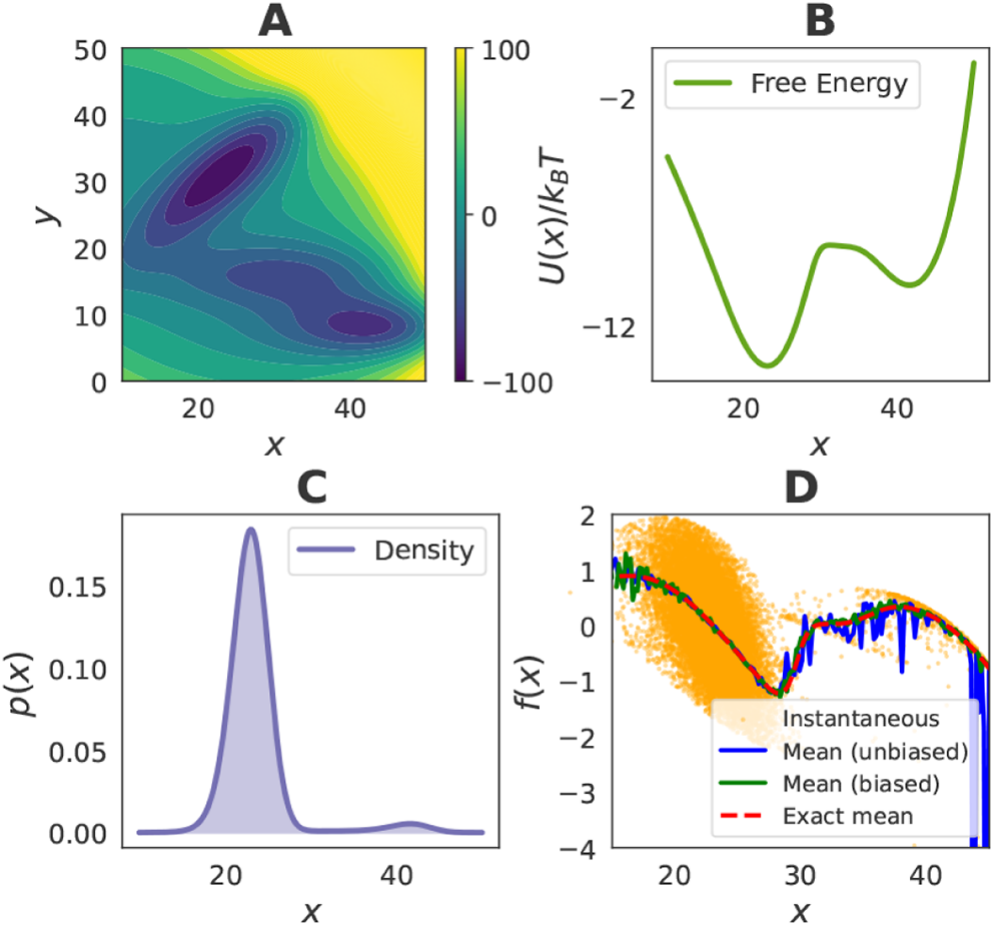
Finite data size effects in the low-dimensional Müller–Brown system. (A) Two-dimensional Müller-Brown potential energy surface (functional form given in the Supporting Information). (B) Exact free-energy profile along the *x*-axis. (C) Marginal probability density along the *x*-axis. (D) Instantaneous force samples from the unbiased data set projected onto the *x*-axis, shown together with the exact mean force and bin-averaged estimates from biased and unbiased data sets of equal size. The unit of the force is *k*_*B*_ *T*.

Coarse-graining is defined here as projection onto the *x*-axis. The corresponding CG PMF is given exactly by

18 $$\frac{U\left(x\right)}{k_{B}T}=-\ln\int_{-\infty }^{\infty }\exp\left(-\beta u\left(x,y\right)\right)dy$$

with the result shown in Figure 2B. The associated probability density along *x* is $p_{\mathrm{CG}}\left(x\right)=Z_{x}^{-1}\exp\left(-\beta U\left(x\right)\right)$ as shown in Figure 2C. The exact mean force along *x* is obtained from the derivative -*dU*(*x*)/*dx* and is plotted in Figure 2D.

To generate training data, we performed two types of simulations. First, an unbiased trajectory is run until equilibrium, sampling the Boltzmann distribution *p*(**r**) ∝ exp­(-β*u*(**r**)). Second, a biased trajectory is generated using umbrella sampling, with a Gaussian restraint *w*(**r**) applied close to the barrier region to enhance transitions between metastable basins. This simulation samples the biased distribution *p*_*W*_(**r**) ∝ exp­(-β­(*u*(**r**) + *w*(**r**))).

For each configuration **r** generated in either simulation, we record the positions **r**, the unbiased forces **f**(**r**) = −∇*u*(**r**), and the biased forces **f̂**(**r**) = −∇(*u*(**r**) + *w*(**r**)). In the biased case, we also compute importance weights ω­(**r**) = exp­(β*w*(**r**)), which allow reweighting to recover unbiased equilibrium averages. Specifically, any observable ϕ­(**r**) can be estimated by self-normalized importance sampling $E_{p}\left[\phi \left(r\right)\right]\approx \overset{K}{\underset{i=1}{\sum }}\mathrm{\omega ̅}\left(r_{i}\right)\phi \left(r_{i}\right)$ , with $\mathrm{\omega ̅}\left(r_{i}\right)=\omega \left(r_{i}\right)/\overset{K}{\underset{j=1}{\sum }}\omega \left(r_{j}\right)$ , where the sum runs over the *K* configurations sampled from the biased trajectory.

#### Finite Data Size Effects

Sampling from the unbiased equilibrium distribution results in a highly uneven coverage: Most configurations accumulate in the left minimum, while other basins are rarely visited (Figure 2C). This imbalance is further illustrated in Figure 2D, which shows 20,000 instantaneous force samples projected onto the *x* axis, with bin averages used to approximate the mean force. In regions with dense sampling, such as the left basin, the estimated mean force agrees closely with the exact result. In contrast, poorly sampled regions, particularly the right minimum, yield noisy and inaccurate estimates.

This behavior highlights a general limitation of equilibrium simulations with high-energy barriers: finite data sets provide imbalanced and incomplete coverage, and force matching suffers as a result. Biased sampling provides a natural solution: as shown in Figure 2D, bin-averaged mean forces from biased data sets of equal size recover the correct mean force profile with substantially reduced variance. This empirically demonstrates that enhanced sampling alleviates finite data size effects, a challenge that becomes even more pronounced in higher dimensional systems.

#### Unbiased Mean Forces

We next verify that the recomputed mean force remains unbiased if and only if the bias is applied along the coarse-grained degree of freedom. To this end, we generated three data sets with biasing potentials applied along *x*, *y*, and (*x*, *y*), each containing sufficient samples to accurately estimate the mean force (Figure 3). When the bias is applied only along *x*, the mean force profile along *x* is correctly recovered after recomputing the forces with respect to the unbiased potential, without the need for reweighting (Figure 3B). In contrast, when the bias acts along *y* or jointly along (*x*, *y*), reweighting is required to recover the correct mean force (Figure 3C–D).

**Figure 3:**
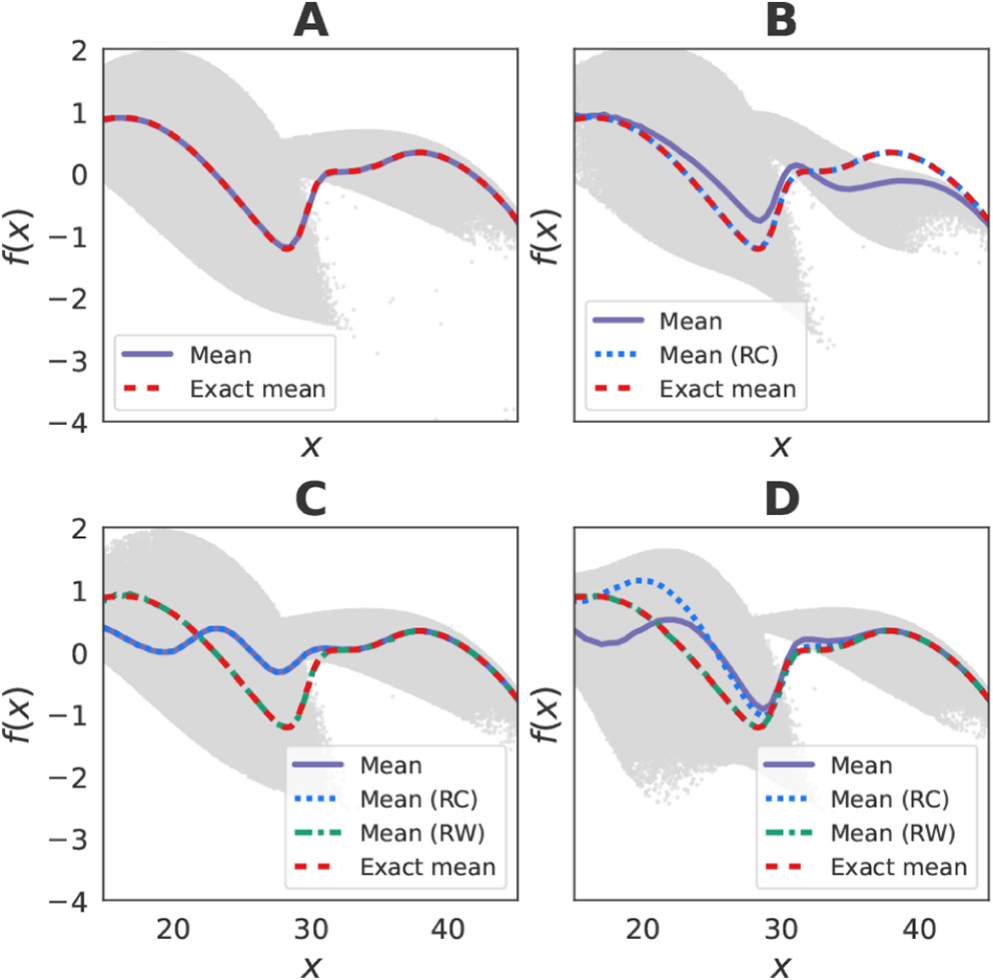
Unbiased mean force recovery in the low-dimensional Müller-Brown system. In all panels, gray dots show instantaneous forces from the corresponding simulations. Overlaid curves denote the exact mean force and bin-averaged estimates: (A) Unbiased simulation. (B) Biased along *x*: bin-averaged estimates include both direct and recomputed (RC) mean forces. (C) Biased along *y*: bin-averaged estimates include direct, RC, and reweighted (RW) mean forces from importance sampling. (D) Biased along (*x*, *y*): bin-averaged estimates include direct, RC, and RW mean forces.

Next, we trained machine learning potentials on both unbiased and biased data sets, restricting the bias to the *x* coordinate, to assess its effect on model accuracy. Potentials are parametrized using a neural network with *radial basis function* (RBF) features as input, followed by several fully connected layers. The RBF layer maps coordinates into a high-dimensional feature space,

19 $$\phi_{j}\left(x\right)=\exp\left(-\frac{{\left(x-c_{j}\right)}^{2}}{2\sigma^{2}}\right),,j=1,...,K$$

where {*c*_*j*_} denote the centers and σ controls the width of the features. These localized features improve the ability of the network to capture nonlinear variations in the mean force landscape compared to using raw coordinates (Supporting Information Figure S1).

Figure 4A reports the mean-squared error (MSE) between the predicted and exact mean force as a function of the amount of training data. Models trained on biased data sets reach lower error and variance with only a few thousand samples, whereas models trained on unbiased data sets require orders of magnitude more data, yet still exhibit larger variance and higher error. Direct comparison of the learned force curves (Figure 4B–C) further illustrates this difference: While both models reproduce the mean force in densely sampled regions, biased training achieves much lower uncertainty and accurately recovers both the overall shape and the fine features of the mean force with limited data. Unbiased training, on the contrary, captures only the broad trend and fails to reproduce local structure even with orders of magnitude more samples. We additionally compare the exact free energy profile with MLP simulations in the Supporting Information (Figure S2), where simulation profiles are obtained via the negative natural logarithm of the sampled position histograms.

**Figure 4:**
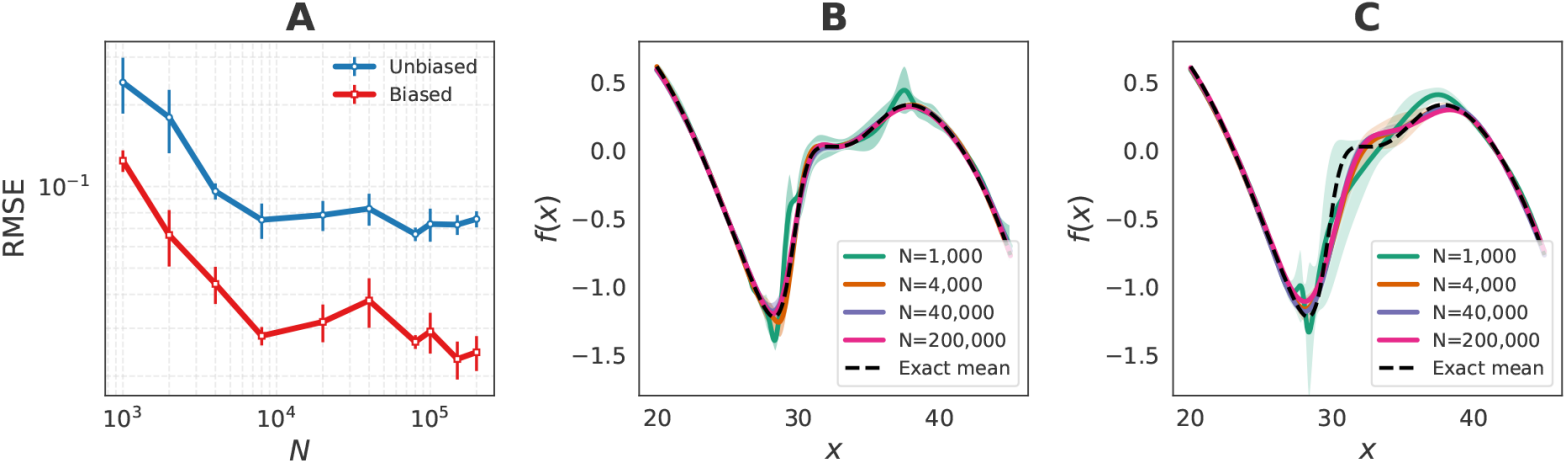
Results for the low-dimensional Müller–Brown potential. (A) Root-mean-square error (RMSE) of predicted forces as a function of the number of training samples (*N*). RMSE values are computed relative to the exact mean force over 500 equally spaced points in the interval *x* ∈ [20, 45]. Results are shown for models trained on biased data sets generated with umbrella sampling and on unbiased data sets. Error bars represent the standard deviation across five independently trained models with different random seeds. (B) Exact mean force compared with model-predicted forces trained on biased data sets obtained via umbrella sampling. *N* indicates the number of training samples; uncertainties reflect variations across five independently trained models. (C) Same as (B), but using unbiased data sets for training.

### Coarse Graining of Capped Alanine in Water

As for the molecular benchmark, we demonstrate our approach on the coarse-graining of solvated capped alanine (alanine dipeptide), a prototypical system for conformational transitions. The coarse-grained mapping retains all ten heavy atoms while discarding hydrogens and water molecules, as illustrated in Figure 5A.

**Figure 5:**
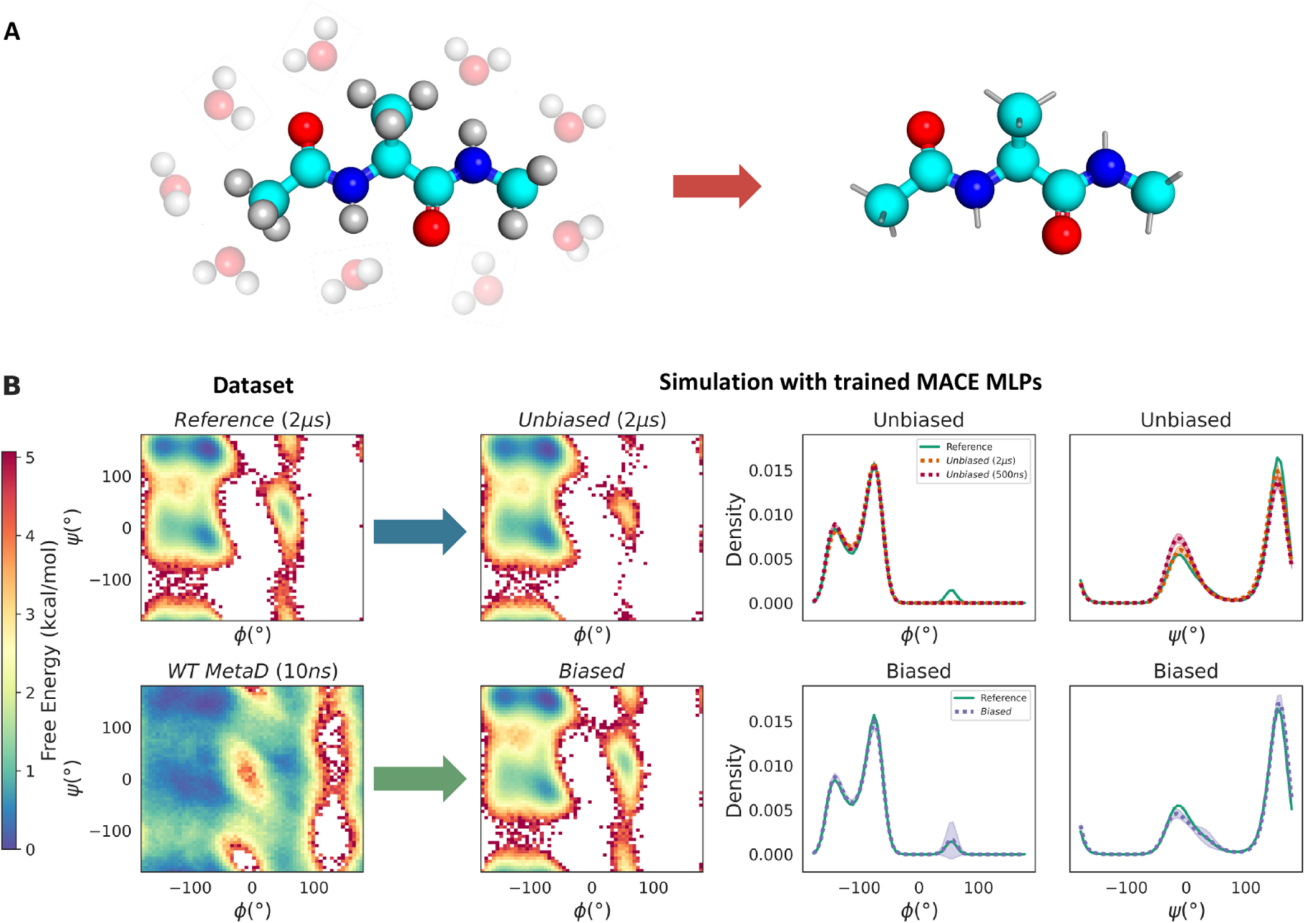
Coarse-graining mapping of capped alanine and the resulting free energy profiles. (A) Mapping from the all-atom solvated model (left) to the coarse-grained (CG) model retaining the ten heavy backbone atoms (right). (B) Free-energy surfaces and one-dimensional dihedral distributions for data sets and CG model simulations. The left column (“Dataset”) shows the reference 2 μs unbiased MD free-energy surface and the well-tempered metadynamics (WT MetaD, 10 ns) data set used for model training. The right columns (“Simulation with trained MACE MLPs”) show the corresponding free-energy surfaces and one-dimensional ϕ/ψ dihedral distributions obtained from CG simulations using models trained on the respective data sets. Mean values and standard deviations (shaded regions) are computed from 100 independent CG trajectories of 100 ns each.

We generate both unbiased and biased data sets for training. The unbiased data set is obtained from a 500 ns MD trajectory at 300 K, from which 5 × 10^5^ configurations are sampled uniformly in time. Biased data sets are generated using well-tempered metadynamics (WT MetaD) with backbone dihedrals ϕ (C–N–Cα-C) and ψ (N–Cα–C-N) as collective variables, employing PLUMED^72^ with GROMACS.^78^ Simulations are performed with bias factors γ = 1.5, 3, 6, 9, each of length 10 ns, and 5 × 10^5^ samples are collected per data set. The Ramachandran plots corresponding to these data sets are shown in the Supporting Information (Figure S6). As γ increases, the simulations explore progressively larger regions of conformational space, particularly transition regions between metastable basins. To explicitly validate the quality of the sampling, we employed two practical convergence criteria: (i) the time-evolution of the biased collective variables to ensure rapid mixing between states, and (ii) the stability of the reconstructed free energy profiles over time across independent replicas. As detailed in the Supporting Information (Figures S3–S4), the biased simulations exhibit frequent transitions and thermodynamic convergence (stabilization of Δ*F*) within few nanoseconds. In contrast, the unbiased reference simulation fails to achieve comparable convergence even after 500 ns due to rare transition events. A sufficiently long 2 μs unbiased MD trajectory is generated as a reference. For all biased simulations, instantaneous forces are recomputed with respect to the unbiased potential using the rerun feature of GROMACS.

To illustrate the effects of finite data size and mean force invariance in the molecular system, we consider a generalized coordinate *q*, such as a dihedral angle of the backbone θ. The conjugate force is

20 $$Q_{\theta }=\underset{i}{\sum }f_{i}\cdot \frac{\partial r_{i}}{\partial \theta }$$

where **f**_*i*_ = -*∂u*/*∂* **r**_*i*_ is the Cartesian force on atom *i* and *∂* **r**_*i*_/*∂*θ is its displacement under a unit change in θ. *Q*_θ_ represents the generalized torque that drives the rotation around the dihedral. As shown in the Supporting Information Figure S5, mean generalized torque ⟨*Q*_θ_⟩ calculated from unbiased trajectories fluctuates strongly in sparsely sampled transition regions, illustrating the limitations of equilibrium data in capturing the full conformational landscape. In contrast, recomputing forces from biased trajectories yields mean torques with much lower variance and correctly recovers the reference profile, confirming invariance under CG coordinate-dependent bias (eq 10).

We then train the MACE model on these data sets using the chemtrain framework^79,80^ with the same hyperparameter settings (listed in the Supporting Information). CG simulations are performed under Langevin dynamics at 300 K using JAX M.D..^81^ For evaluation, we run 100 independent CG simulations of 100 ns each, initialized from random configurations, for both the unbiased data set and biased data sets with different bias factors γ. Ramachandran plots and Dihedral distributions (Figure 5B) show that models trained on unbiased data fail to recover the metastable basin α_L_ at ϕ ≈ 0^◦^-100^◦^ on the right-hand side of the Ramachandran map, whereas biased training with sufficiently large γ recovers both modes accurately. Quantifying metastable populations across five independent models (Supporting Information Figure S7–S8) show that unbiased data sets and low-γ WT MetaD assign nearly zero probability to the metastable state α_L_, while higher-γ data sets accurately capture it.

Next, we investigate the effect of the size of the training data set. For each, we run 100 independent 100 ns CG simulations and compare the resulting ϕ-ψ distributions to reference MD. Specifically, we compute the KL divergence and mean-squared error (MSE) of the torsional free energy on discrete histograms (Figure 6). For MSE, unbiased data sets initially yield smaller errors as a result of denser sampling of the left-hand mode in the Ramachandran map. However, as the size of the data set increases, biased simulations achieve a lower overall MSE by accurately reproducing both modes. For KL divergence, biased data sets consistently outperform the 500 ns unbiased data set, and surpass the 2 μs unbiased data set when more samples are used, as they capture the global free-energy landscape more faithfully. Together, these results highlight the better accuracy of training on biased data sets.

**Figure 6:**
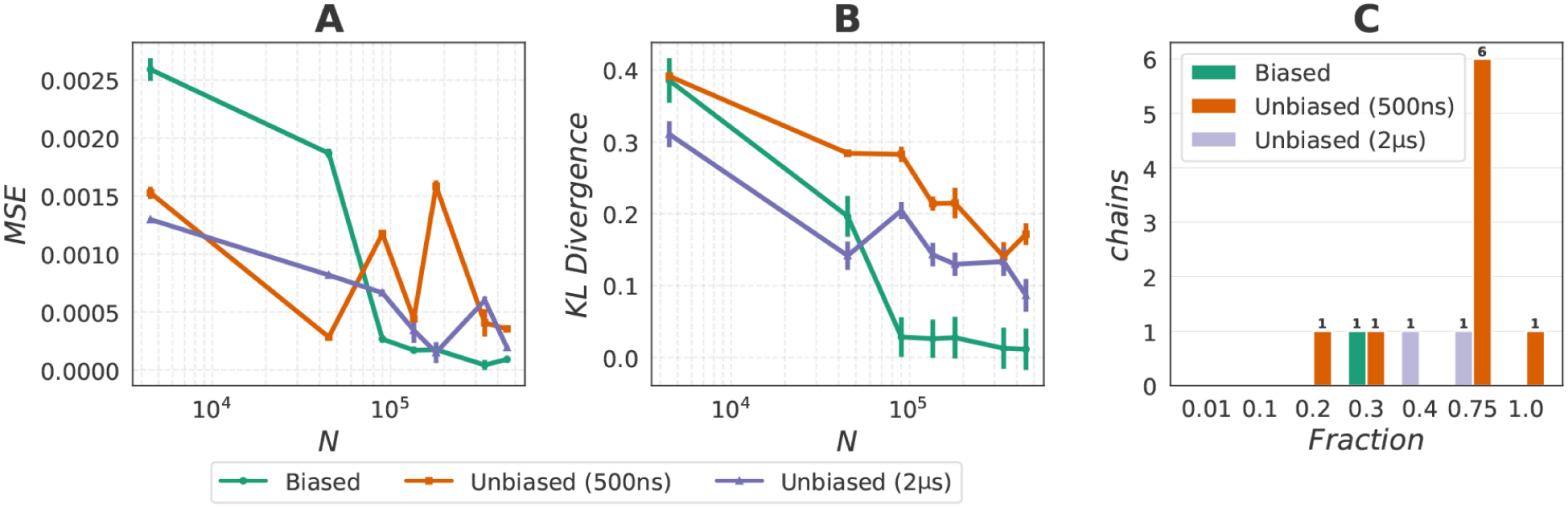
Model accuracy and stability for capped alanine. (A) Mean squared error (MSE) between discrete free energies on the ϕ/ψ plane for varying training data sizes. Mean and standard deviation are estimated from 100 trajectories of 100 ns each. (B) Kullback–Leibler (KL) divergence between discrete free energies on the ϕ/ψ plane for varying training data sizes. Mean and standard deviation are again computed from 100 trajectories of 100 ns each. (C) Number of unstable trajectories as a function of training data size, expressed as a fraction of the total samples. Numbers indicate how many out of 100 trajectories are unstable; if not specified, all trajectories are stable.

Finally, we assess stability by monitoring numerical instabilities across training fractions (Figure 6C). Chains are considered unstable and removed when the predicted potential energy reaches unphysically high values. In previous analyses, these unstable chains were already excluded; here we explicitly report their occurrence. Most simulations remain stable across 100 ns, with failures occurring only in a few chains. For the 500 ns unbiased data set, up to six chains diverge at fraction 0.75, with isolated failures at fractions 0.2, 0.3, and 1.0. The 2 μs unbiased data set shows single-chain failures at fractions 0.4 and 0.75. In contrast, biased data sets exhibit only one failure at fraction 0.3. These results indicate that biased data sets improve both accuracy and stability by providing broader coverage of configuration space. For completeness, we report free energy surfaces without chain removal as well as per-chain results (Supporting Information, Figure S9–S10). Additionally, to assess computational efficiency, we present a wall-clock time comparison between our MACE model and the reference ATMD simulations performed in GROMACS (Supporting Information, Table A2).

## Conclusion

Our work introduces enhanced sampling as a principled strategy for generating training data and improving the efficiency of training CG MLPs within the force matching framework. We show that mean forces are invariant under biases applied along CG degrees of freedom once the forces are recomputed, enabling biased trajectories to be used directly for training without reweighting. Using umbrella sampling and well-tempered metadynamics as representative enhanced sampling methods, we demonstrate on both the Müller-Brown potential and capped alanine that biased data sets provide substantially improved force coverage and data efficiency, yielding accurate and stable CG models without the need for physics-based priors.

Our results demonstrate that enhanced sampling provides a practical solution to the finite data size effects inherent in force matching. By accelerating transitions across energy barriers, enhanced sampling significantly reduces data generation time. It also enriches the training data set with configurations that are rarely visited in unbiased simulations. As a result, neural networks can reconstruct the potential of mean force with higher accuracy, particularly in transition regions. Notably, this improvement is achieved without introducing additional physical priors: enhanced sampling itself supplies the necessary regularization. In this way, it functions as a data-side regularizer, allowing complex CG interactions to be learned directly from data, while reducing the dependence on hand-crafted corrections.

One limitation of our approach is its dependence on prior knowledge of collective variables (CVs) or reaction coordinates suitable for biasing. In the Müller-Brown and capped alanine benchmarks, the relevant slow modes are well understood, allowing the bias to be applied directly to the coarse-grained degrees of freedom. However, for more complex biomolecular systems, it is challenging to identify such CVs.^82–84^ When chosen CVs do not adequately capture the slow dynamics, enhanced sampling may not sufficiently enrich force coverage, limiting the improvement of the resulting models. Machine learning techniques for automated reaction coordinate discovery^85–89^ provide a potential solution, and their integration could facilitate a broader application to high-dimensional CG mappings and larger biomolecules.

Looking ahead, the framework allows for several natural extensions. Biasing could be applied not only along predefined collective variables, but also along arbitrary coarse-grained degrees of freedom, including learned slow coordinates. Additional enhanced sampling methods, such as adaptive biasing force,^90–92^ replica exchange or tempering,^7^ variationally enhanced sampling,^93,94^ could readily be integrated to further improve efficiency and transferability. Furthermore, one could relax the requirement of biasing only the mapped coordinates by applying bias to orthogonal degrees of freedom; this would necessitate recovering unbiased mean forces via explicit reweighting strategies^95,96^ (e.g., via Markov state models^97,98^ ). Such extensions share conceptual roots with established frameworks for transferring potentials between thermodynamic states.^35,99–101^ However, the benefit of biasing orthogonal degrees of freedom is likely limited in this context, since coarse-grained mappings are typically designed to explicitly capture the slowest, most relevant coordinates.

Additionally, an active learning cycle alternating between model training, uncertainty quantification of mean forces, and targeted bias placement would enable systematic sampling of regions with high uncertainty, producing data sets that are both efficient and informative.^36,102,103^ The balance between biased and unbiased simulations can also be optimized, for example, by employing pretraining-finetuning paradigms that take advantage of complementary data sources.^104^ From a practical perspective, our approach could be used to construct or refine large-scale data sets for training transferable CG MLPs,^105,106^ improving transferability across varying thermodynamic conditions and chemical compositions. It could also provide information for generative models that typically lack force supervision^107^ or support energy-based neural samplers.^61,108,109^ Overall, we believe that our method represents a fundamental advance over current methodologies and opens new opportunities to tackle outstanding challenges for efficient learning of coarse-gained molecular models.

## Supplementary Material



## Data Availability

The code used in this work is publicly available on GitHub at https://github.com/tummfm/biased-force-matching and https://github.com/tummfm/chemtrain.

## Notes

Details of data set generation, training procedures, hyperparameters, computational cost, and additional qualitative results on molecular systems (PDF)
