# Supplementary material for: Enhanced Sampling for Efficient Learning of Coarse-Grained Machine Learning Potentials
Source: J Chem Theory Comput. 2025 Dec 24;22(1):219–30. doi: 10.1021/acs.jctc.5c01712 (PMC12805510; doi:10.1021/acs.jctc.5c01712)
Supplement: Supplementary file 1 [file ct5c01712_si_001.pdf]

Supporting Information for:

Enhanced Sampling for Efficient Learning of Coarse-Grained Machine Learning Potentials

Weilong Chen,[†] Franz Görlich,[†] Paul Fuchs,[†] and Julija Zavadlav[*,†,‡]

†*Professorship of Multiscale Modeling of Fluid Materials, Department of Engineering Physics and Computation, TUM School of Engineering and Design, Technical University of Munich, 80333 Munich, Germany*

‡*Atomistic Modeling Center (AMC), Munich Data Science Institute (MDSI), Technical University of Munich, 85748 Garching, Germany*

E-mail: julija.zavadlav@tum.de

Supporting Information

Invariance of the Force Matching Minimum under Biased Sampling

Here we demonstrate that minimizing the Force Matching error over a biased distribution $p_W(\mathbf{r})$ yields the same optimal force field as minimizing it over the unbiased equilibrium distribution $p_{\text{AT}}(\mathbf{r})$, provided the bias potential W depends only on the coarse-grained coordinates $\xi(\mathbf{r})$.

Let $\mathbf{F}_\theta(\mathbf{R}) \equiv -\nabla U(\mathbf{R};\theta)$ be the coarse-grained model force and $\mathbf{f}(\mathbf{r})$ be the unbiased atomistic force. The general loss function for the biased sampling distribution $p_W(\mathbf{r})$ is:

$$\chi^2_W(\theta) = \int d\mathbf{r}\, p_W(\mathbf{r}) \left\| \mathbf{F}_\theta(\xi(\mathbf{r})) - \xi(\mathbf{f}(\mathbf{r})) \right\|^2. \tag{1}$$

We decompose the biased probability density $p_W(\mathbf{r})$ into the marginal distribution of the CG coordinates $p_W(\mathbf{R})$ and the conditional distribution $p_W(\mathbf{r}|\mathbf{R})$:

$$\chi^2_W(\theta) = \int d\mathbf{R}\, p_W(\mathbf{R}) \underbrace{\left[\int d\mathbf{r}\, p_W(\mathbf{r}|\mathbf{R}) \left\| \mathbf{F}_\theta(\mathbf{R}) - \xi(\mathbf{f}(\mathbf{r})) \right\|^2 \right]}_{I_W(\mathbf{R})}. \tag{2}$$

Note that inside the conditional integral $I_W(\mathbf{R})$, the mapping $\xi(\mathbf{r})$ is constrained to $\mathbf{R}$. This makes the model force $\mathbf{F}_\theta(\xi(\mathbf{r}))$ equal to the constant vector $\mathbf{F}_\theta(\mathbf{R})$, which can be factorized out of the inner integral. Expanding the squared norm in $I_W(\mathbf{R})$:

$$\begin{aligned} I_W(\mathbf{R}) = \|\mathbf{F}_\theta(\mathbf{R})\|^2 &\int d\mathbf{r}\, p_W(\mathbf{r}|\mathbf{R}) \\ &- 2\mathbf{F}_\theta(\mathbf{R}) \cdot \int d\mathbf{r}\, p_W(\mathbf{r}|\mathbf{R})\, \xi(\mathbf{f}(\mathbf{r})) \\ &+ \int d\mathbf{r}\, p_W(\mathbf{r}|\mathbf{R})\, \|\xi(\mathbf{f}(\mathbf{r}))\|^2. \end{aligned} \tag{3}$$

Using the normalization $\int p_W(\mathbf{r}|\mathbf{R}) d\mathbf{r} = 1$ and defining the conditional expectation, this

simplifies to:

$$I_W(\mathbf{R}) = \|\mathbf{F}_\theta(\mathbf{R})\|^2 - 2\mathbf{F}_\theta(\mathbf{R}) \cdot \langle \xi(\mathbf{f}(\mathbf{r})) \rangle_{p_W|\mathbf{R}} + \langle \|\xi(\mathbf{f}(\mathbf{r}))\|^2 \rangle_{p_W|\mathbf{R}}. \tag{4}$$

As shown in the main text, the conditional distribution is invariant to the bias: $p_W(\mathbf{r}|\mathbf{R}) = p(\mathbf{r}|\mathbf{R})$. Consequently, the conditional expectation of the force is the true thermodynamic mean force, $\mathbf{F}^*(\mathbf{R})$:

$$\langle \xi(\mathbf{f}(\mathbf{r})) \rangle_{p_W|\mathbf{R}} = \mathbf{F}^*(\mathbf{R}). \tag{5}$$

We can now "complete the square" for the term $I_W(\mathbf{R})$ with respect to the model parameters θ:

$$I_W(\mathbf{R}) = \left\|\mathbf{F}_\theta(\mathbf{R}) - \mathbf{F}^*(\mathbf{R})\right\|^2 + \sigma^2_{\text{noise}}(\mathbf{R}), \tag{6}$$

where $\sigma^2_{\text{noise}}(\mathbf{R}) = \langle \|\xi(\mathbf{f})\|^2 \rangle_{p_W|\mathbf{R}} - \|\mathbf{F}^*(\mathbf{R})\|^2$ is the intrinsic variance of the atomistic forces, which is independent of θ.

Substituting this back into the global loss function:

$$\chi^2_W(\theta) = \int d\mathbf{R}\, p_W(\mathbf{R}) \left\|\mathbf{F}_\theta(\mathbf{R}) - \mathbf{F}^*(\mathbf{R})\right\|^2 + C, \tag{7}$$

where C is a constant independent of θ. This form reveals that the objective function is strictly minimized when:

$$\mathbf{F}_\theta(\mathbf{R}) = \mathbf{F}^*(\mathbf{R}) \tag{8}$$

for all $\mathbf{R}$ where $p_W(\mathbf{R}) > 0$.

While the integral is weighted by the biased marginal $p_W(\mathbf{R})$ (which differs from the unbiased marginal $p_{\text{CG}}(\mathbf{R})$), the function $\mathbf{F}_\theta(\mathbf{R})$ that minimizes the error is identical in both cases.

Low-dimensional Müller Brown Potential Experiments

Potential Parameters For the two-dimensional test system, the Müller–Brown potential is defined as[1]

$$U(x, y) = U_1(x, y) + U_2(x, y) + U_3(x, y) + U_4(x, y), \tag{9}$$

with

$$\begin{aligned}
U_1(x, y) &= -17.3 \exp\left[-0.0039(x-48)^2 - 0.0391(y-8)^2\right], \\
U_2(x, y) &= -8.7 \exp\left[-0.0039(x-32)^2 - 0.0391(y-16)^2\right], \\
U_3(x, y) &= -14.7 \exp\left[-0.0254(x-24)^2 + 0.043(x-24)(y-32) - 0.0254(y-32)^2\right], \\
U_4(x, y) &= \ \ 1.3 \exp\left[0.00273(x-16)^2 + 0.0023(x-16)(y-24) + 0.00273(y-24)^2\right].
\end{aligned}$$

Biasing Potentials For umbrella sampling, we introduce below biasing potentials. For one-dimensional bias along the x coordinate, we use[2]

$$W_x(x) = -4 \exp\left[-\frac{(x-32.0)^2}{2 \cdot 5^2}\right].$$

For one-dimensional bias along the y coordinate, we use

$$W_y(y) = 6 \exp\left[-\frac{(y-30.0)^2}{2 \cdot 4^2}\right].$$

For two-dimensional biasing in both x and y, we use

$$W_{xy}(x, y) = 10 \exp\left[-\frac{(x-25.0)^2 + (y-25.0)^2}{2 \cdot 5^2}\right].$$

Simulation Parameters. For the Müller–Brown experiments, Langevin dynamics is used with the parameters summarized in Table A1.

Table A1: Parameters used in Langevin simulations of the Müller–Brown potential.

Time step (Δt)	0.1
Number of steps per trajectory	10^7
Number of trajectories	10
Mass (m)	1.0
Friction coefficient (γ)	0.1
Temperature ($k_{\mathrm{B}}T$)	1.0
Initial positions	Uniform in $[10, 50]^2$
Initial velocities	Gaussian with $\sigma = 0.1$
Downsampling interval	Every 10 steps

Data Generation. Langevin dynamics is performed in two dimensions according to

$$m\ddot{\mathbf{r}} = -\nabla(u(\mathbf{r}) + W(\mathbf{r})) - \gamma m\dot{\mathbf{r}} + \sqrt{2\gamma k_{\mathrm{B}}Tm}\,\boldsymbol{\eta}(t),$$

where $u(x, y)$ is the Müller–Brown potential, $W(x, y)$ is the applied bias, γ is the friction coefficient, and $\boldsymbol{\eta}(t)$ is Gaussian white noise. Ten independent trajectories of length 10^7 steps are generated, with initial positions drawn uniformly from the box $[10, 50]^2$ and initial velocities sampled from a Gaussian distribution scaled by 0.1. All samples are recorded every 10 steps. For each saved configuration, both biased forces (from $\nabla(u + W)$) and unbiased forces (from ∇u) are computed. Importance weights are obtained as $\exp(\beta W(\mathbf{r}))$.

Training Hyperparameters. The low-dimensional models are trained on MB datasets using a radial basis function (RBF) feature map followed by a multilayer perceptron (MLP). The model architecture consists of an RBF expansion with $K = 100$ centers (initialized uniformly in $[10, 50]^2$ and optimized during training) and a fixed width $\sigma = 5.0$. The RBF features are passed through four fully connected layers of size 128 with softplus activation, followed by a linear output layer. Training is performed with the Adam optimizer (via Optax) at a constant learning rate of 10^{-4} and a batch size of 128. For each dataset size $N \in \{10^3, 2\times10^3, 4\times10^3, 8\times10^3, 2\times10^4, 4\times10^4, 8\times10^4, 10^5, 1.5\times10^5, 2\times10^5\}$, training is continued until a fixed budget of 30,000 gradient updates is reached, such that the number

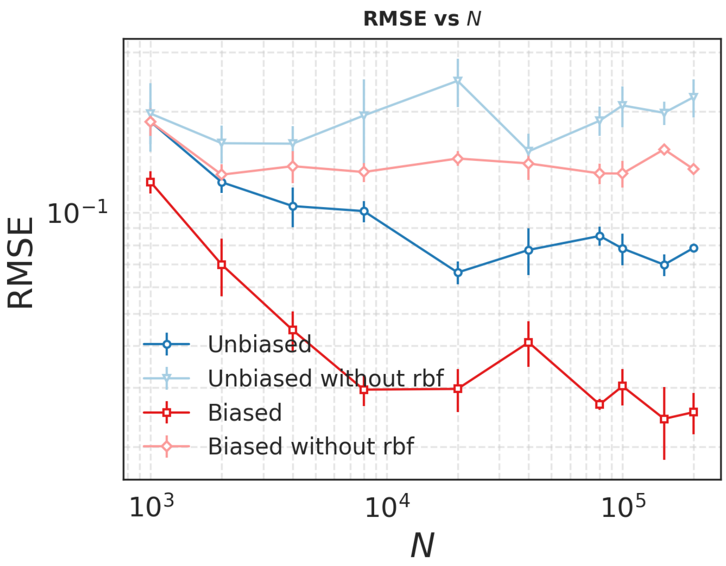

Figure S1: **Force prediction accuracy as a function of training set size N.** Shown are root mean square errors (RMSE) of predicted forces for biased and unbiased models, with and without radial basis function (RBF) features. Error bars denote standard deviations across five independent training runs with different random seeds.

of epochs varied with N. To estimate uncertainity, five independent models are trained for each dataset size with different random seeds. In addition, an ablation study is carried out by training models with and without the RBF feature map, as shown in Figure S1, in order to assess the contribution of the RBF representation to force prediction accuracy.

Free Energy Surface Figure S2 compares the free energy profiles along the x-coordinate obtained from the MLP simulations against the exact analytical reference. The exact free energy is calculated by integrating the Boltzmann factor of the Müller-Brown potential over the orthogonal y-degree of freedom. For the simulation-derived profiles, we computed the normalized probability density $P(x)$ by histogramming the x-coordinates from the unbiased and biased MLP trajectories (excluding the initial equilibration phase). The free energy is then estimated via $U(x) = -k_B T \ln P(x)$.

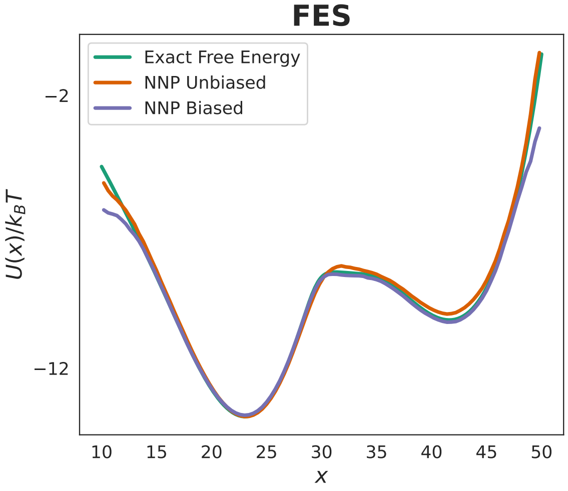

Figure S2: **Free energy profiles for the Müller-Brown potential.** The exact reference is computed by numerical integration over the orthogonal coordinate. Profiles for the MLP models is estimated from trajectory histograms.

Alanine Dipeptide Experiments

Unbiased Data Generation The system is parameterized with the AMBER99SB-ILDN force field[3] and solvated in a cubic box of TIP3P water with side length 3.7 nm. The simulation is carried out using `GROMACS`.[4] After energy minimization, the system is equilibrated under both NVT and NPT ensembles to stabilize density and temperature at 300 K. Production dynamics are performed in the NVT ensemble using a velocity-rescale thermostat (time constant 0.1 ps) with separate coupling groups for solute and solvent. Long-range electrostatics are treated with particle mesh Ewald (PME), and van der Waals interactions are truncated at 1.0 nm. The simulation is run with a 2 fs timestep, and forces, coordinates, and energies are saved every 1 ps, yielding a dataset of 500,000 configurations. Heavy-atom trajectories are extracted, centered, and corrected for periodic boundary conditions for subsequent analysis.

Well-tempered Metadynamics Data Generation To enhance sampling of transition regions, we perform well-tempered metadynamics [5] simulations of alanine dipeptide in explicit solvent using `GROMACS` coupled with `PLUMED`.[6] The dihedral angles ϕ (C–N–C$_\alpha$–C) and

ψ (N–C$_\alpha$–C–N) are chosen as collective variables (CVs). Gaussian hills of height 1.2 kJ/mol and width 0.35 rad are deposited every 500 integration steps (1 ps). Four datasets are generated with different bias factors $\gamma = 1.5, 3, 6, 9$. Each simulation is run for 5×10^6 steps (10 ns) with a time step of 2 fs, while positions and forces are recorded every 10 steps (0.02 ps), resulting in 5×10^5 saved configurations per dataset. To ensure unbiased training labels, all forces are recomputed by rerunning the saved trajectories in `GROMACS` without the metadynamics bias. This is achieved using the `mdrun -rerun` functionality, where the stored trajectories (`md.trr`) are re-evaluated against the unbiased force field (`md.tpr`) to obtain the correct atomic forces. For example:

```
gmx mdrun -s md.tpr -rerun md.trr -deffnm rerun_forces
```

This guarantees that each configuration yields forces corresponding to the underlying unbiased potentials, independent of the applied metadynamics bias.

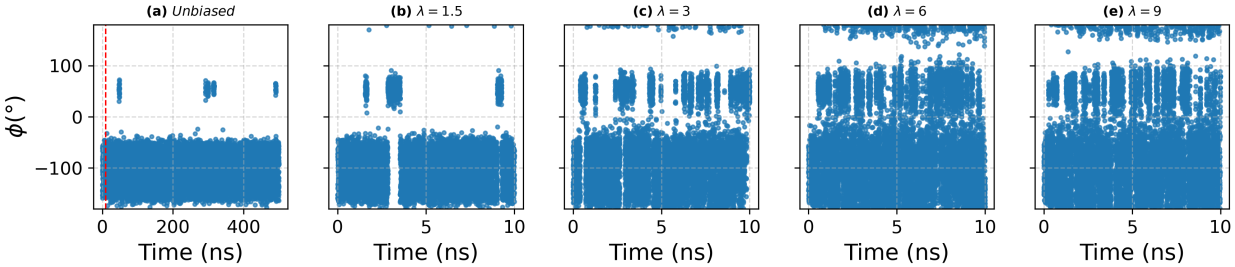

Figure S3: **Time evolution of the ϕ angle shown as scatter plots.** (a) Unbiased 500 ns simulation. The red vertical line at 10 ns indicates that the mode with $\phi \in [0°, 100°]$ has not yet been visited. (b–e) Biased simulations of 10 ns each with different values of γ.

Convergence of MD Simulations We firstly visualize the time evolution of the ϕ dihedral angle. Figure S3 shows the trajectory of ϕ as a function of simulation time. In the unbiased case, the system remains trapped in its initial basin and requires nearly 50 ns before it first crosses into the right mode of ϕ. In contrast, the biased simulations rapidly promote transitions between modes, with frequent crossings observed within just 10 ns.

To assess the convergence properties of unbiased and biased simulations, we quantify convergence by monitoring the free energy difference between distinct metastable modes as

a function of simulation time. As shown in Figure S4, the unbiased simulation does not converge after 500 ns due to the slow transition. In contrast, the biased simulations with bias factor $\gamma = 6$ display rapid convergence: across five independent trajectories, the free energy differences stabilize within approximately 2 ns. These results highlight the substantial acceleration of sampling and convergence achieved by well-tempered metadynamics relative to unbiased molecular dynamics.

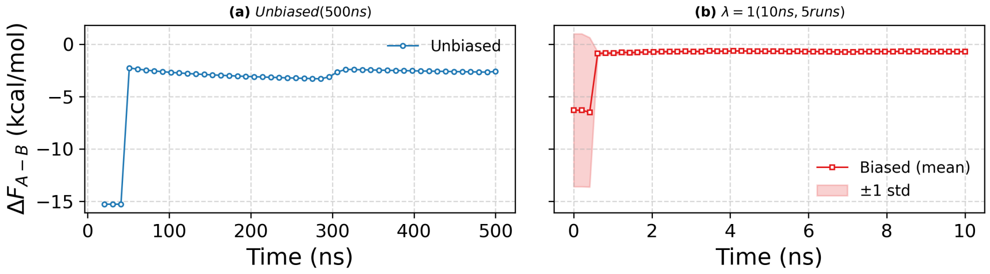

Figure S4: **Free energy difference between two basins.** (a) Estimate from a single unbiased 500 ns trajectory, which does not converge within this timeframe. (b) Estimate from well-tempered metadynamics with $\gamma = 6$, showing convergence within 10 ns. Uncertainties are obtained from five independent 10 ns simulations initialized with different random velocities.

Unbiased Mean Torques For a generalized coordinate q (e.g., a dihedral angle θ), the conjugate force is the generalized torque

$$Q_\theta = \sum_i \mathbf{f}_i \cdot \frac{\partial \mathbf{r}_i}{\partial \theta},$$

which represents the component of atomic forces driving rotation about the dihedral. As shown in Figure S5, mean torques $\langle Q_\theta \rangle$ computed from unbiased trajectories display large fluctuations in sparsely sampled transition regions. By contrast, recomputing torques from biased trajectories with respect to the unbiased potential recovers the correct mean profile with reduced variance, confirming the invariance of mean forces under coordinate-dependent bias.

Effect of Bias Factors γ on Conformational Sampling. Supplementary Figure S6 illustrates the Ramachandran plots of the training datasets (first row) alongside the coarse-grained MLP simulations trained on each dataset (second row). As the bias factor γ increases, the datasets progressively explore broader regions of conformational space, including transition regions between metastable basins, and the corresponding CG models reproduce these features with increasing fidelity. Supplementary Figure S7 shows the one-dimensional ϕ and ψ distributions, further demonstrating that models trained on higher-γ datasets more accurately capture the reference populations and metastable states.

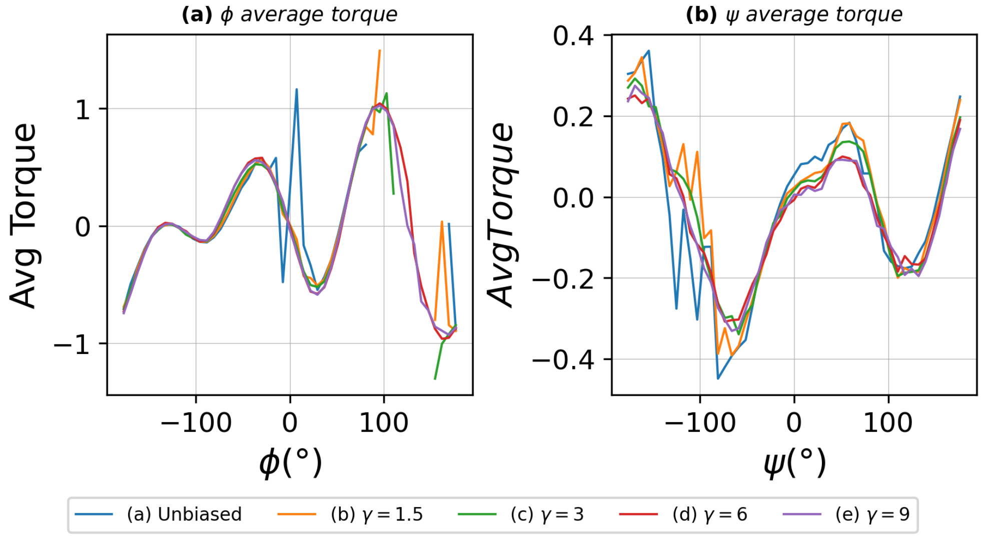

Figure S5: **Dihedral average torques of alanine dipeptide.** From the unbiased and well-tempered reference simulations. Torques are obtained by differentiating the dihedral angles with respect to atomic positions and projecting the instantaneous atomic forces onto these directions, yielding the effective torque driving dihedral rotation. γ is the biasing factor.

Metastable State Populations. As shown in Figure S8, models trained on unbiased data or low-γ WT-MetaD forces assign nearly zero probability to the right-hand metastable state at $\phi \in [0°, 100°]$. Training on higher-γ datasets restores the correct populations, underscoring the advantage of enhanced sampling for force matching.

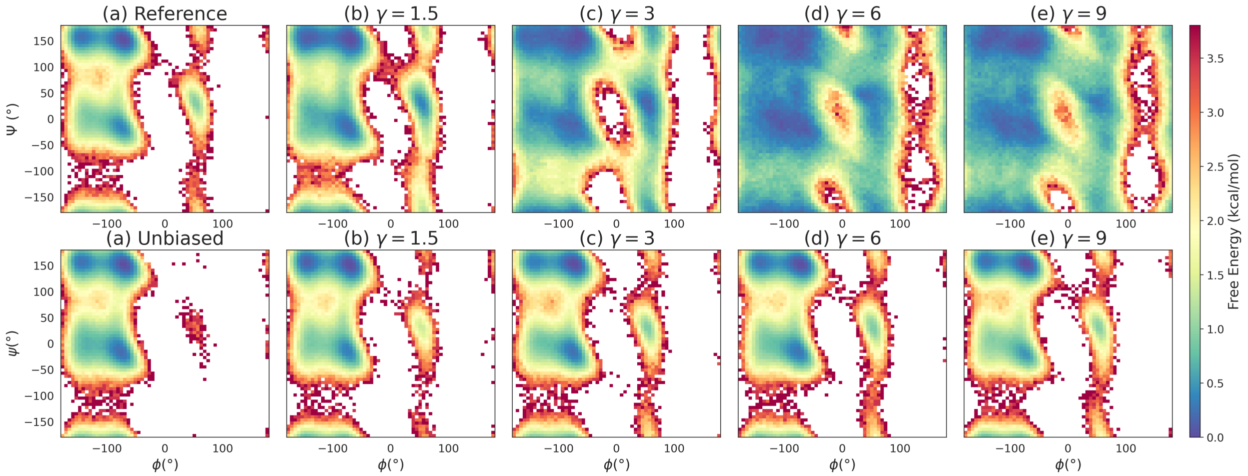

Figure S6: **Ramachandran plots for capped alanine.** First row: results from atomistic MD datasets. (a) Unbiased 2 μs simulation. (b–e) Well-tempered metadynamics simulations with the corresponding γ values. Second row: results from coarse-grained MLP simulations trained on different datasets. (a) Model trained on an unbiased 500 ns dataset. (b–e) Models trained on biased 10 ns datasets. Obtained from simulations with 100 independent 100 ns trajectories.

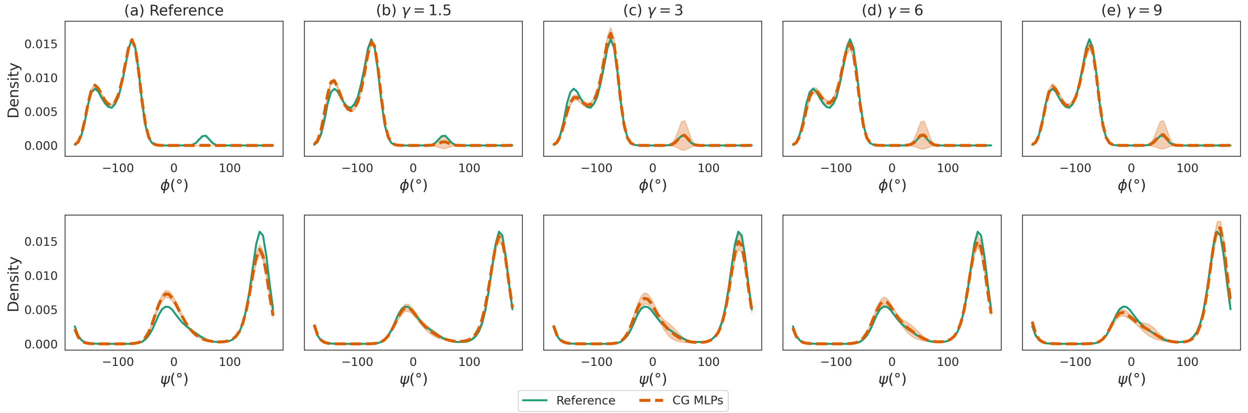

Figure S7: **Probability density distributions of dihedral angles for capped alanine.** First row: distributions of ϕ. Second row: distributions of ψ. Each plot compares the atomistic reference with coarse-grained MLP simulations. Uncertainty is reported as the mean $\pm$ standard deviation over 100 independent 100 ns trajectories.

Training Hyperparameters The CG MLPs are trained by force matching using the `chemtrain` framework[7,8] with the equivariant graph neural network MACE.[9] Training and validation splits are generated with a 90/10 ratio. Neighbor lists are constructed using JAX-MD sparse partitioning with a cutoff of 0.5 nm. All simulations are subsequently run with JAX, M.D.[10] under `chemtrain`.

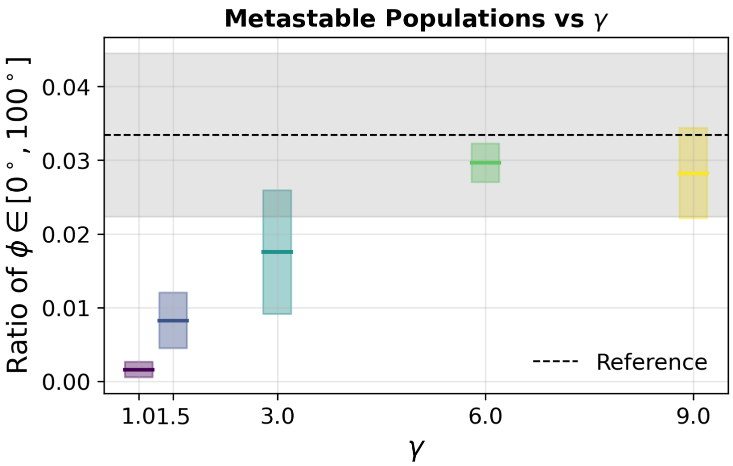

Figure S8: **Probability that ϕ lies within $0°–100°$,** as a function of γ in well-tempered metadynamics. The reference distribution is obtained from a 2 μs unbiased simulation, divided into four folds to estimate mean and variance. For each γ, results are based on 100 independent 10 ns trajectories, with uncertainty estimated across five models trained with different random seeds.

The MACE model is parameterized with hidden irreducible representations of $32 \times 0e + 32 \times 1o$, a readout multilayer perceptron of $16 \times 0e$, and a scalar energy output representation of $1 \times 0e$. The angular momentum expansion is truncated at $\ell_{\text{max}} = 3$, and two message-passing interaction layers are used with correlation order 3. A periodic displacement function ensure proper treatment of boundary conditions.

Training is performed using the force-matching loss and the Adam optimizer with exponential learning rate decay ($\eta_0 = 10^{-3}$, decay rate 0.9), a batch size of 32, and a total of 200 epochs. The parameters used for simulation are those corresponding to the minimum validation loss. Gradients are clipped to a global norm of 1.0. All model training and simulations are performed on a single NVIDIA A100 GPU.

Computational Efficiency To assess the computational performance of our approach, we benchmark the wall-clock time of the coarse-grained simulation against the reference atomistic molecular dynamics. The atomistic simulation is performed using the GPU-accelerated version of `GROMACS` with the same solvation parameters used for dataset generation (4957 atoms total), utilizing a time step of 0.5 fs. The coarse-grained MACE simulation is executed

using JAX-MD on the reduced 10-atom system with a time step of 2.0 fs.

As summarized in Table A2, the coarse-grained MACE model achieves a comparable raw throughput for a single trajectory. Note, however, that this single-trajectory benchmark does not fully utilize the parallel capabilities of the A100 GPU. In practice, we run multiple trajectories in parallel (batching) to fully saturate the GPU, yielding approximately an order of magnitude increase in aggregate throughput. Furthermore, the coarse-grained nature of the model allows for larger integration time steps (e.g., 4–6 fs for capped alanine) without stability issues. As reported in recent work,[11] the combination of these factors can yield effective sampling speedups of up to two orders of magnitude compared to classical atomistic MD. Crucially, while the computational cost of the CG MLP is comparable to classical force fields, its accuracy is not bound by empirical parameterization. By training on data generated from high-fidelity atomistic ML potentials (e.g., at the DFT level), the CG model can achieve quantum-chemical accuracy in regimes where standard classical force fields are structurally or chemically limited. Furthermore, the trained CG MLP can serve as a standalone, differentiable energy function. This allows it to be used as an energy predictor for reweighting tasks, providing practical utility without the need for performing simulations.

Table A2: **Wall-clock time comparison between Atomistic MD and CG MACE.** Benchmarks were performed on a single NVIDIA A100 GPU. The atomistic system includes explicit solvent (4957 atoms), while the coarse-grained system contains only the 10 heavy atoms.

**Method**	**Atoms**	**Timestep** (fs)	**Time / ns** (s)	**Performance** (ns/day)
ATMD (GROMACS)	4957	0.5	309.94	278.76
CG MACE (JAX-MD)	10	2.0	296.16	291.30

Stability of CG MLP Simulations Figure S9 shows an example of the free-energy surfaces computed for each chain individually; the model is trained on the full unbiased dataset. In this case, chain number 24 is identified as the unstable chain due to the predicted potential energy reaching unphysical values. Supplementary Figure S10 compares the free-energy surfaces of all chains before and after removing chain 24.

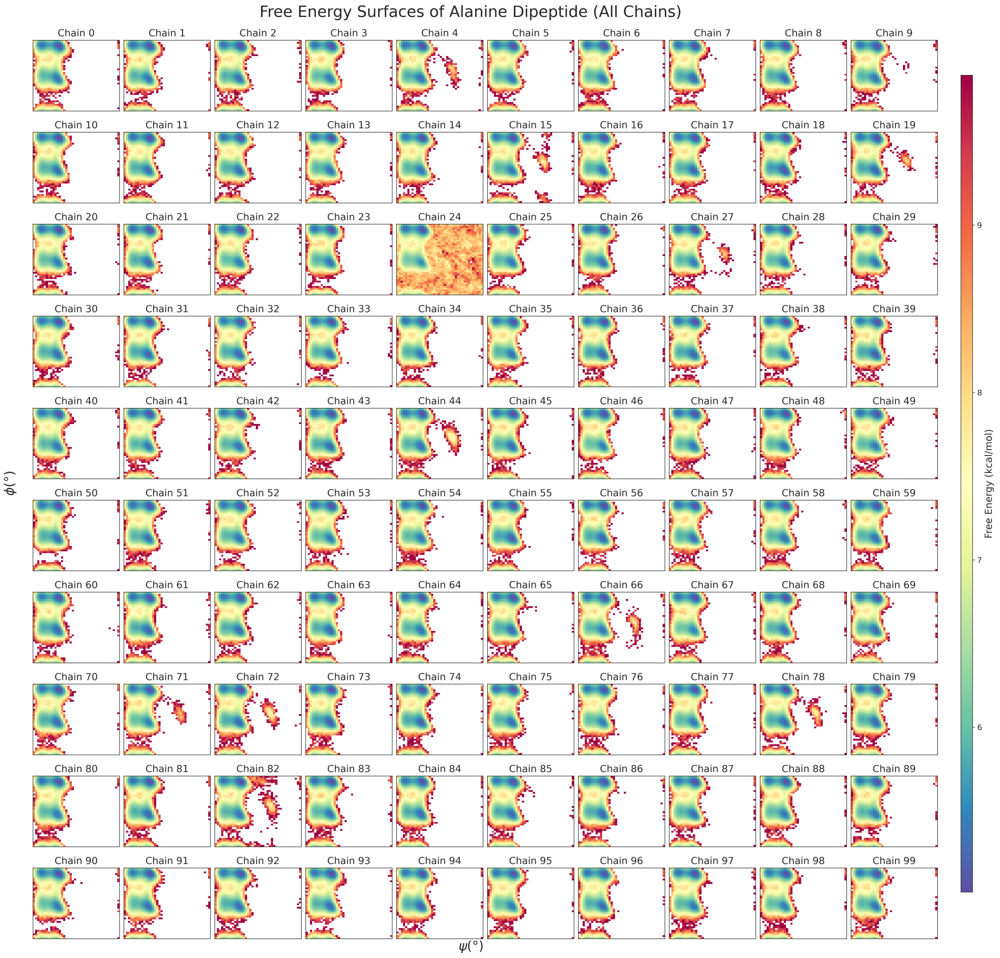

Figure S9: **Free energy surfaces for 100 independent 100 ns simulations.** Each chain is shown individually; chain 24 exhibits instability.

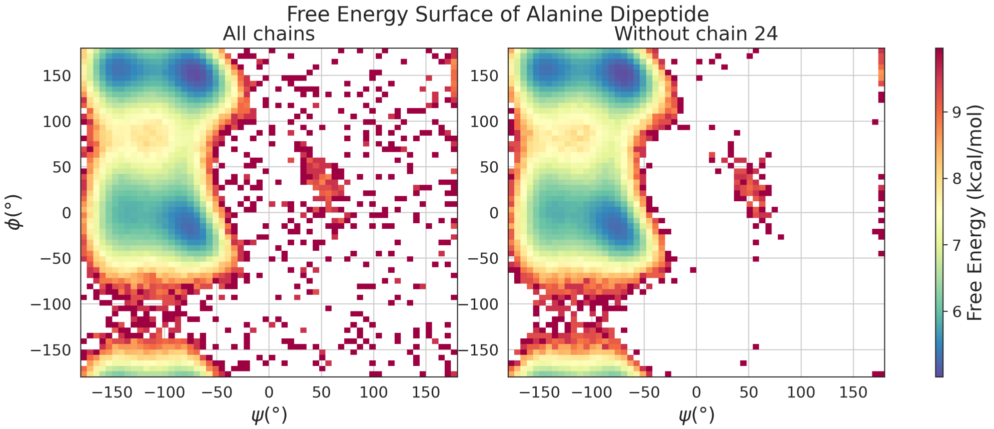

Figure S10: **Free energy surfaces of alanine dipeptide.** (left) Results including all chains. (right) Results after removing unstable chain number 24.

References

(1) Raja, S.; Šípka, M.; Psenka, M.; Kreiman, T.; Pavelka, M.; Krishnapriyan, A. S. Action-Minimization Meets Generative Modeling: Efficient Transition Path Sampling with the Onsager-Machlup Functional. *arXiv preprint arXiv:2504.18506* **2025**,

(2) Torrie, G.; Valleau, J. Nonphysical sampling distributions in Monte Carlo free-energy estimation: Umbrella sampling. *Journal of Computational Physics* **1977**, *23*, 187–199.

(3) Lindorff-Larsen, K.; Piana, S.; Palmo, K.; Maragakis, P.; Klepeis, J. L.; Dror, R. O.; Shaw, D. E. Improved side-chain torsion potentials for the Amber ff99SB protein force field. *Proteins: Structure, Function, and Bioinformatics* **2010**, *78*, 1950–1958.

(4) Van Der Spoel, D.; Lindahl, E.; Hess, B.; Groenhof, G.; Mark, A. E.; Berendsen, H. J. GROMACS: fast, flexible, and free. *Journal of computational chemistry* **2005**, *26*, 1701–1718.

(5) Barducci, A.; Bussi, G.; Parrinello, M. Well-tempered metadynamics: a smoothly converging and tunable free-energy method. *Physical review letters* **2008**, *100*, 020603.

(6) Bonomi, M.; Branduardi, D.; Bussi, G.; Camilloni, C.; Provasi, D.; Raiteri, P.; Donadio, D.; Marinelli, F.; Pietrucci, F.; Broglia, R. A.; others PLUMED: A portable plugin for free-energy calculations with molecular dynamics. *Computer Physics Communications* **2009**, *180*, 1961–1972.

(7) Fuchs, P.; Chen, W.; Thaler, S.; Zavadlav, J. chemtrain-deploy: A parallel and scalable framework for machine learning potentials in million-atom MD simulations. *Journal of Chemical Theory and Computation* **2025**, *21*, 7550–7560.

(8) Fuchs, P.; Thaler, S.; Röcken, S.; Zavadlav, J. chemtrain: Learning deep potential models via automatic differentiation and statistical physics. *Computer Physics Communications* **2025**, *310*, 109512.

(9) Batatia, I.; Kovacs, D. P.; Simm, G.; Ortner, C.; Csányi, G. MACE: Higher order equivariant message passing neural networks for fast and accurate force fields. *Advances in neural information processing systems* **2022**, *35*, 11423–11436.

(10) Schoenholz, S.; Cubuk, E. D. Jax md: a framework for differentiable physics. *Advances in Neural Information Processing Systems* **2020**, *33*, 11428–11441.

(11) Charron, N. E.; Bonneau, K.; Pasos-Trejo, A. S.; Guljas, A.; Chen, Y.; Musil, F.; Venturin, J.; Gusew, D.; Zaporozhets, I.; Krämer, A.; others Navigating protein landscapes with a machine-learned transferable coarse-grained model. *Nature Chemistry* **2025**, 1–9.
